# High-Temperature Deformation Behaviour of UNS S32750 Super Duplex Stainless Steel (SDSS) Alloy

**DOI:** 10.3390/ma17215151

**Published:** 2024-10-23

**Authors:** Vasile Dănuț Cojocaru, Nicolae Șerban, Elisabeta Mirela Cojocaru, Nicoleta Zărnescu-Ivan

**Affiliations:** Faculty of Materials Science and Engineering, National University of Science and Technology Politehnica of Bucharest, 060042 Bucharest, Romania; dan.cojocaru@upb.ro (V.D.C.); nicolae.serban@upb.ro (N.Ș.); elisabeta.cojocaru@upb.ro (E.M.C.)

**Keywords:** super duplex stainless steel (SDSS) alloys, microstructural characterisation, plasticity

## Abstract

In this study, the high-temperature deformation behaviour of the UNS S32750 Super Duplex Stainless Steel (SDSS) alloy was investigated by means of deformability and microstructure evolution in the (1050–1200) °C temperature (T) range. The deformability of the UNS S32750 SDSS alloy was investigated by the up-setting method using a gravity-drop hammer, with the following deformation energy/impact energy ($E^{*}$): 545.2 J, 1021.5 J, 1480.6 J, and 1905.3 J. Data referring to deformation resistance ($\sigma_{c}^{\prime }$) and mechanical work ($A^{*}$) as a function of deformation temperature (T) and deformation energy/impact energy ($E^{*}$) were obtained and analysed. It was shown that increasing the deformation temperature leads to an increase in the obtained deformation degree (degree of reduction in height). By analysing the rate of increase in the deformation degree as a function of the applied impact energy, it was shown that the rate of increase in the deformation degree rises with the increase in the applied impact energy. Also, it was observed that the evolution of the deformation resistance ($\sigma_{c}^{\prime }$) as a function of temperature (T) shows a decreasing tendency while increasing the deformation temperature for all impact energies and that the evolution of the mechanical work ($A^{*}$) as a function of temperature (T) shows a decreasing tendency while increasing the deformation temperature for all impact energies. The microstructure evolution of the UNS S32750 SDSS alloy was investigated by X-ray diffraction (XRD) and Scanning Electron Microscopy-Electron Backscatter Diffraction (SEM-EBSD) techniques. It was observed that, in all cases, the microstructure shows intensely deformed grains, strongly elongated in the rolling direction in both ferrite (δ) and austenite (γ) intensely deformed grains. The intensity of grain deformation is increasing with the increase in the applied deformation degree. Also, it was observed that increasing the deformation temperature leads to a strong increase in the weight fraction of the dynamically recrystallised (DRX) ferrite (δ) grains.

## 1. Introduction

Super Duplex Stainless Steel (SDSS) alloys are considered ideal materials to operate in many aggressive/corrosive environments. Such environments are usually found in petrochemical and power plant applications. SDSS alloys showed a unique combination of strength, toughness, and resistance to pitting, crevice corrosion, and stress corrosion cracking [1,2,3,4,5,6,7,8]. Those unique properties are owed to their two-phase microstructure, which contains approximately equal volume fractions of BCC (body-centred cubic) ferrite (δ) and FCC (face-centred cubic) austenite (γ) phases [9,10,11]. Usually, the ferrite (δ) phase is constituted as the matrix phase and is responsible for the strength and stress corrosion cracking resistance, while the austenite (γ) phase shows an island-like in the matrix phase and is responsible for the toughness, ductility, and uniform corrosion resistance [12,13,14,15].

The coexistence of these two phases, ferrite (δ) and austenite (γ), each having different characteristics, such as different crystal structures and stacking fault energy (SFE) values, with very different responses to external stresses at high temperatures and with different dynamic softening processes, would highly increase the difficulty in deformation of SDSSs at elevated temperatures [16,17,18,19,20,21]. Hence, it is necessary to understand the hot deformation behaviour of the SDSS alloys in order not to cause the appearance of macrostructural and microstructural defects and to optimise the hot deformation process parameters to improve their plasticity.

It is known that the austenite (γ) phase, being very ductile and having a high SFE value, tends to soften by dynamic recovery (DRV), in contrast to the ferrite (δ) phase, which is harder and has a low SFE value, undergoes dynamic recrystallisation (DRX) during hot deformation [22,23,24,25]. Apart from the different deformation modes of those two constituent phases, the ferrite (δ) and austenite (γ) phase balance could change with the deformation temperature due to the occurrence of the ferrite (δ) to austenite (γ) phase transition, increasing the volume fraction of the ferrite (δ) phase and decreasing the volume fraction of the austenite (γ) phase as the temperature increases, resulting in worsening of the mechanical and corrosion resistance properties [26,27,28,29,30]. Therefore, the hot deformation process parameters (deformation temperature, applied deformation degree, and applied strain rate) are important factors in controlling the plasticity of SDSS alloys. These hot deformation process parameters are essential in all metal hot working and need to be studied and controlled, thus avoiding the appearance of defects or degradation of final products by applying an inappropriate thermomechanical treatment.

The experiments performed in this study aim to establish the high-temperature deformation behaviour of the UNS S32750 Super Duplex Stainless Steel (SDSS) alloy in the temperature (T) range of (1050–1020) °C through both deformability and microstructure evolution by finding the finest values for the obtained deformation degree and by studying the main microstructural modifications that happen.

## 2. Materials and Methods

**Figure 1:**
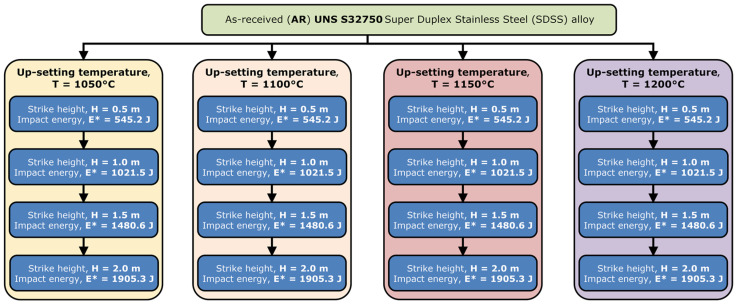
Schematic representation of the applied processing route to investigate the deformability of the UNS S32750 SDSS alloy.

**Figure 2:**
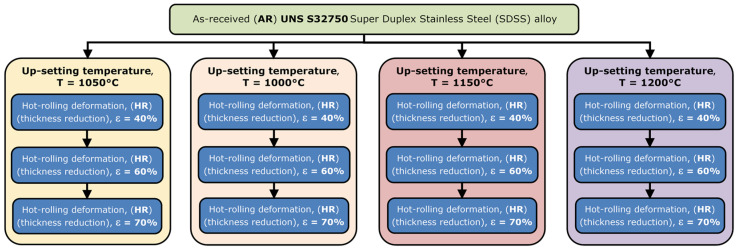
Schematic representation of the applied processing route to investigate the microstructure evolution during deformation by rolling of the UNS S32750 SDSS alloy.

The investigated UNS S32750/EN 1.4410/F53 SDSS alloy (BHG Edelsthal Freital GmbH, Freital, Germany) was thermomechanically processed in order to investigate the alloy’s deformability (Figure 1) and microstructure evolution during hot deformation by rolling (Figure 2).

Figure 1 shows the schematic representation of the applied thermomechanical (TM) processing route in order to investigate the alloy’s deformability. The investigation method was represented by up-setting using a gravity-drop hammer with a mass of 117 kg and strike heights between 0.5 m and 2.0 m. The following investigation parameters were considered: obtained deformation degree (degree of reduction in height) (ε), deformation resistance ($\sigma_{c}^{\prime }$), and mechanical work ($A^{*}$) as a function of the deformation temperature (T) and deformation energy/impact energy (E*). The as-received (AR) UNS S32750 SDSS alloy samples, which were cylinder-shape samples with a l/d = 1.5 ratio (Ø 20 × 30 mm), were subjected to up-setting deformation at temperatures between 1050 °C and 1200 °C and impact energies between 545.2 J (strike hight: 0.5 m) and 1905.3 J (strike hight: 2.0 m).

Figure 2 shows the schematic representation of the applied thermomechanical (TM) processing route in order to investigate the microstructure evolution during deformation by rolling. The investigation method was represented by the rolling using an Ø 200 × 200 mm roughing rolling mill. The following investigation parameters were considered: applied deformation degree (degree of reduction in thickness) (ε) as a function of the deformation temperature (T). The AR UNS S32750 SDSS alloy samples, which were rectangular-shape samples with dimensions of 30 × 10 × 100 mm (b × h × l), were subjected to deformation by rolling at temperatures between 1050 °C and 1200 °C and applied deformation degrees between 40% and 70%.

All TM processed specimens were investigated by X-ray diffraction (XRD) and Scanning Electron Microscopy-Electron Backscatter Diffraction (SEM-EBSD) techniques from a microstructural point of view in order to observe the microstructural constituent phases and occurred changes induced during TM processing. The metallographic preparation procedure applied to all samples is presented and detailed elsewhere [31].

The identification of the constituent phases was carried out by X-ray diffraction using a RIGAKU MiniFlex600 (RIGAKU, Tokyo, Japan) benchtop diffractometer by observing the alloy’s patterns between 2θ = 30° and 2θ = 100° in 2θ using Cu-Kα radiation, with a limit of detection of 0.1 to 1 wt. % for each phase. The evolution of the microstructure was carried out by the SEM-EBSD technique using a Tescan Vega III–LMU (TESCAN, Brno, Czech Republic) SEM coupled with a BRUKER Quantax e-Flash 1000 (Bruker Corporation, Billerica, MA, USA) EBSD detector.

## 3. Results and Discussion

### 3.1. As-Received UNS S32750 Super Duplex Stainless Steel Alloy

Before experiencing high-temperature deformation, the AR UNS S32750 SDSS alloy was microstructurally analysed. The alloy’s microstructural analysis was performed using X-ray diffraction and SEM-EBSD investigation techniques. The results of the XRD diffraction analysis are illustrated in Figure 1, where it can be seen clearly that the alloy consists of two phases, namely ferrite (δ) and austenite (γ) phases, without another phase being found in the microstructure of the AR UNS S32750 SDSS alloy.

**Figure 3:**
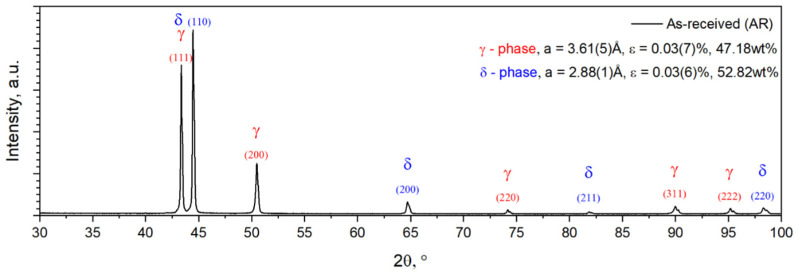
The XRD diffraction pattern of the as-received UNS S32750 SDSS alloy.

As shown in Figure 3, the ferrite (δ) phase is indicated by (110), (200), (211), and (220) diffraction lines, while the austenite (γ) phase is indicated by (111), (200), (220), (311), and (222) diffraction lines. The Rietveld analysis for the ferrite (δ) and austenite (γ) phases found in the microstructure revealed approximate values of the lattice parameter of a = 2.88(1) Å for the δ phase and a = 3.61(5) Å for the γ phase. The calculated internal average micro-strain (ε) was situated close to 0.03(6) % for the δ phase and close to 0.03(7) % for the γ phase. Also, the analysis revealed a weight fraction close to 52.82 wt% for the δ phase and a weight fraction close to 47.18 wt% for the γ phase.

**Figure 4:**
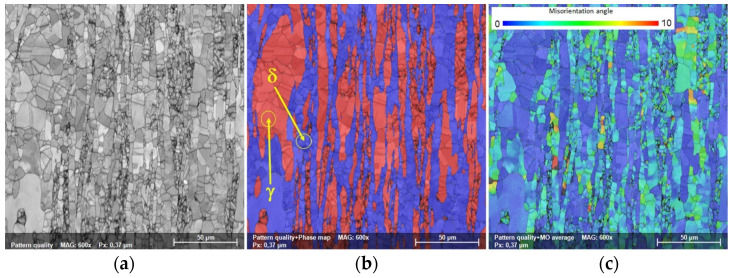
The SEM-EBSD microstructure of the as-received UNS S32750 SDSS alloy (**a**), distribution map of ferrite (δ) and austenite (γ) phases (**b**), and average Modal Orientation (MO) distribution map of both ferrite (δ) and austenite (γ) phases (**c**).

Figure 4 shows typical SEM-EBSD images of the AR UNS S32750 SDSS alloy, where one can view a typical SEM-EBSD microstructure image (Figure 4a), the distribution map of ferrite (δ) and austenite (γ) phases (Figure 4b), and the average Modal Orientation (MO) distribution map of both the ferrite (δ) and austenite (γ) phases (Figure 4c). Similar to the observations made by the XRD analysis, the SEM-EBSD analysis showed the presence only of ferrite (δ) and austenite (γ) phases in the distribution map image (Figure 4b). The austenite (γ) phase, coloured in red, shows elongated grain morphology within the ferrite (δ) phase coloured in blue, which serves as a matrix.

The volume fraction of constituent phases was quantified using an SEM-BSE analysis and showed that the content of the ferrite (δ) phase was situated close to 55.06 wt%, while the content of the austenite (γ) phase was situated close to 44.94 wt%. One can notice that, in comparison with the XRD analysis, the SEM-EBSD analysis showed a larger content of ferrite (δ) phase and a lower content of austenite (γ) phase, the difference being approximately 2.24 wt%, which resides from the specificity of the investigation techniques (i.e., the XRD analyses are performed on areas measuring mm^2^, while the SEM-EBSD is performed on areas measuring μm^2^, different measurement resolution, etc.).

The Modal Orientation (MO) distribution map can be used as a visualisation tool at the microstructural level to assess the residual strain–stress field resulting during thermomechanical processing [32,33]. The MO distribution map shows grains with deviations from the average grain orientation, deviations that occur during thermomechanical processing due to the accumulated strain–stress field induced by the slip/twinning, dynamic recrystallisation, strain hardening, etc. [34,35].

The distribution map of the average Modal Orientation (MO) is presented in Figure 4c, which shows that both ferrite (δ) and austenite (γ) phases have grains showing a relatively low misorientation, with a maximum misorientation close to 9° recorded in the case of the ferrite (δ) phase, while the austenite (γ) phase grains show a much lower misorientation. The relatively low misorientation of the AR UNS S32750 Super Duplex Stainless Steel alloy indicates a small probability of micro-crack appearance/development during thermomechanical processing.

**Figure 5:**
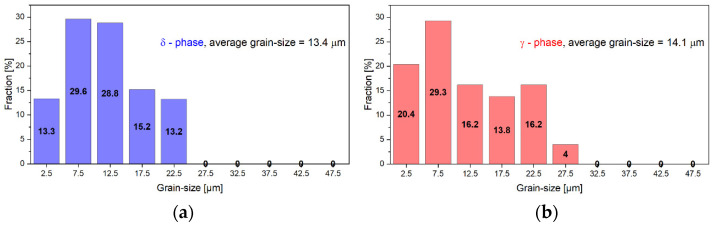
Grain-size distribution of ferrite (δ) phase (**a**) and austenite (γ) phase (**b**) in the as-received UNS S32750 SDSS alloy.

The grain-size analysis of both the ferrite (δ) and austenite (γ) phases was performed using the SEM-EBSD technique. Figure 5 shows the grain-size distribution of the ferrite (δ) (Figure 5a) phase and austenite (γ) phase (Figure 5b). In the case of both phases, one can observe a narrow grain-size distribution, with a grain size up to approximately 27 μm and an average grain size of the ferrite (δ) phase grains close to 13.4 μm and the average grain size of the austenite (γ) phase grains close to 14.1 μm.

### 3.2. High-Temperature Deformation Behaviour

#### 3.2.1. Deformability of UNS S32750 Super Duplex Stainless Steel Alloy

The material’s plasticity can be understood as the ability of materials to deform plastically, i.e., to change their original shape under the action of an external stress field, without destroying their integrity (without the appearance of integrity defects such as microcracks, cracks, fissures, etc.). The main factors influencing the plasticity of materials are the following: the material’s phase structure and thermo-mechanical processing conditions (deformation temperature, applied stress, strain rate, etc.).

Since plastic deformation processes are very different in terms of the mechanical deformation scheme and the conditions under which different factors influence the plasticity of materials undergoing deformation, no universally valid method of determination has been found so far, and, as such, there is no single quantity that gives an absolute and true value for plasticity. For this reason, plasticity is estimated using several indirect methods, more or less characteristic of the concrete conditions of plastic deformation processes, such as up-setting, tensile tests, torsion tests, etc. The values, thus obtained, can only be used to compare the deformation behaviour of some materials with others.

The up-setting method is the most widely used experimental method for determining plasticity (the permissible degree of deformation) because it has the following advantages: simplicity of operation, the stress state diagram and strain rate are practically the same as those obtained in real cases of plastic deformation by forging/close-die forging (forging and close-die forging being the most widely used methods of plastic deformation applied on an industrial scale in the production of parts), it can be carried out at strain rates required by those used in real technologies, and, at the same time, it makes it possible not only to find the optimum range of plastic deformation temperatures but also to determine the plasticity (permissible degree of deformation).

The experimental program for determining the plasticity (the permissible degree of deformation) involved carrying out up-setting experiments using a gravity-drop hammer at four constant impact energies. The up-setting experiments were carried out using a gravity-drop hammer with a mass of 117 kg and four different drop/strike heights: 0.5 m, 1.0 m, 1.5 m, and 2.0 m.

The impact energy of the gravity-drop hammer can be expressed using the following relation:

(1) $$E^{*}=m\cdot g\cdot H\cdot \eta \;\left[J\right]$$

where m is the mass of the falling part of the gravity-drop hammer [kg]; g is the gravitational acceleration [m/s^2^]; H is the falling height of the gravity-drop hammer [m]; and η is the yield coefficient [%].

Considering that the yield coefficient depends on the fall height of the gravity-drop hammer, the yield coefficient used in the experimental determinations, determined using Heim’s method, was as follows: η ≈ 95 (for a fall height of H = 0.5 m), η ≈ 89 (for a fall height of H = 1.0 m), η ≈ 86 (for a fall height of H = 1.5 m), and η ≈ 83 (for a fall height of H = 2.0 m).

The deformation degree (degree of reduction in height) obtained after a hammer strike can be expressed by the following relation:

(2) $$\varepsilon =\frac{h_{0}-h_{1}}{h_{0}}\cdot 100\left[\%\right]$$

where h_0_ is the initial height of the sample [mm]; and h_1_ is the final height of the deformed sample [mm].

After performing the thermomechanical processing, all samples were visually investigated in order to assess the appearance of microcracks/cracks on sample surfaces, which indicated the surpassing of the alloy’s permissible degree of deformation. In all cases, no microcracks/cracks were identified, indicating that the thermomechanical processing was performed without reaching the alloy’s permissible degree of deformation.

**Figure 6:**
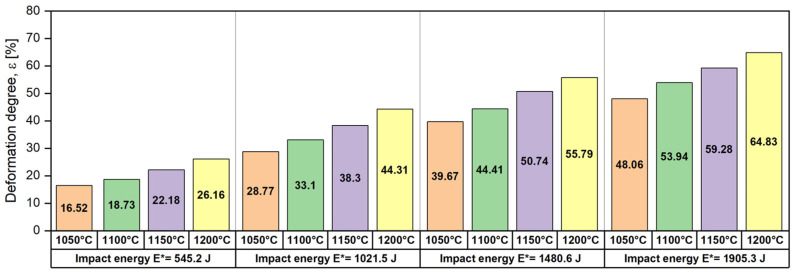
The evolution of deformation degree (ε) as a function of temperature (T) and impact energy (E*) in the case of UNS S32750 SDSS alloy.

Table 1 summarises the obtained data for the deformation degree (ε) of the UNS S32750 SDSS alloy as a function of strike height (H), impact energy (E*), and temperature (T), respectively. Figure 6 illustrates the evolution of the deformation degree (ε) as a function of temperature (T) and impact energy (E*). As can be noted, the deformation degree (ε) has the same increasing tendency for all temperatures, the smallest deformation degrees (ε) being recorded in the case of 1050 °C temperature, followed by 1100 °C, 1150 °C, and 1200 °C temperatures, respectively.

As observed, increasing the deformation temperature leads to an increase in the obtained deformation degree (degree of reduction in height), i.e., at an impact energy of 545.2 J, the following deformation degrees are obtained: 16.52% for a deformation temperature of 1050 °C, 18.73% for a deformation temperature of 1100 °C, 22.18% for a deformation temperature of 1150 °C, and 26.16% for a deformation temperature of 1200 °C. Analysing the rate of increase in the deformation degree as a function of the applied impact energy, one can notice that the rate of increase in the deformation degree is increasing with the increase in the applied impact energy, i.e., the rate of increase in the deformation degree between 1050 °C and 1200 °C for an impact energy of 545.2 J is situated close to 0.06%/°C, while for an impact energy of 1905.3 J, it is situated close to 0.11%/°C, which is an increase of about 74%.

Knowing the resistance to plastic deformation, depending on the nature of the metal material and the thermomechanical processing conditions, is one of the main issues to be taken into account both when choosing the machine/equipment on which plastic deformation will be performed and the range of deformation temperatures.

Since the external deformation force is transmitted to the deformed material by stress acting in the same direction, plastic deformation is sometimes taken to mean the stress required for the deformed material to change from an elastic to a plastic state. The direct measurement of deformation stress is difficult, requiring special, high-performance equipment to determine the optimum range of deformation temperatures, and the deformation resistance of the material at the test temperature is determined from Siebel’s relation, which takes into account the influence of friction and in which the pressure on the tool contact surface is assumed to be uniformly distributed:

(3) $$L=\sigma_{c}^{\prime }\cdot \left(1+\frac{1}{3}\cdot \mu \cdot \frac{d_{1}}{h_{1}}\right)\cdot V_{d}\;\;\;\;\;\;\left[J\right]$$

where $\sigma_{c}^{\prime }$ is the deformation resistance of the material at the test temperature [N/m^2^, Pa]; μ is the coefficient of external friction [%]; $d_{1}$ is the average diameter of the deformed specimen [m]; $h_{1}$ is the height of the deformed specimen [m]; and $V_{d}$ is the volume of material displaced in one impact [m^3^]. In the case of hot up-setting deformation without lubrication, the coefficient of external friction can take values between 0.25 and 0.35.

The volume of material displaced in one strike can be expressed by the following relation:

(4) $$V_{d}=V\cdot \varepsilon \;\;\;\;\;\left[m^{3}\right]$$

where V is the total material volume of the specimen [m^3^]; and ε is the deformation degree (degree of reduction in height) [%].

The average diameter of the deformed specimen can be expressed by the following relation:

(5) $$d_{1}=d_{0}\cdot \sqrt{\frac{h_{0}}{h_{1}}}\;\;\;\;\;\;\left[m\right]$$

The mechanical work can be replaced by the impact energy of the falling part of the free-falling hammer from relation (1) as follows:

(6) $$\;L=E^{*}$$

In this way, the deformation resistance can be determined using the following relation:

(7) $$\sigma_{c}^{\prime }=\frac{E^{*}}{\left(1+\frac{1}{3}\cdot \mu \cdot \frac{d_{1}}{h_{1}}\right)\cdot V\cdot \varepsilon }=\frac{m\cdot g\cdot H\cdot \eta }{\left(1+\frac{1}{3}\cdot \mu \cdot \frac{d_{1}}{h_{1}}\right)\cdot V\cdot \varepsilon }\;\;\;\;\;\;\left[\mathrm{Pa}\right]$$

Sometimes, for simplicity, the deformation resistance, $\sigma_{c}^{\prime }$, is replaced by the mechanical work. In turn, the mechanical work of plastic deformation is determined based on the deformation obtained as a result of the applied impact energy ($E^{*}$). The mechanical work can be expressed by the following relation:

(8) $$A^{*}=\frac{E^{*}}{V_{d}}\;\;\;\;\;\;\left[J/m^{3}\right]$$

In order to determine the variation of the deformation resistance ($\sigma_{c}^{\prime }$) and the mechanical work ($A^{*}$) as a function of temperature (T) and impact energy (E*), the experiments were carried out at four temperatures (1050 °C, 1100 °C, 1150 °C, and 1200 °C) and four impact energies (~545 J, ~1.021 J, ~1.480 J, and ~1.905 J).

Table 2 summarises the obtained data for the evolution of deformation resistance ($\sigma_{c}^{\prime }$) and mechanical work ($A^{*}$) as a function of temperature (T) and impact energy (E*) for the UNS S32750 Super Duplex Stainless Steel alloy.

**Figure 7:**
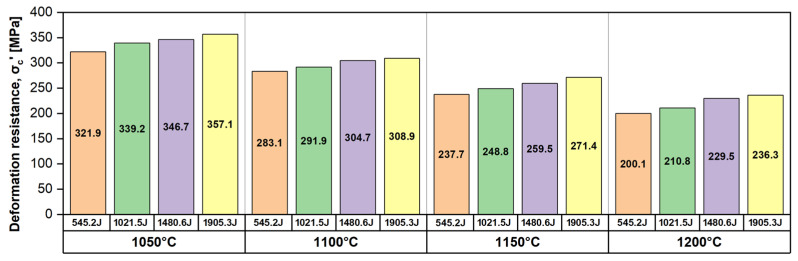
The evolution of deformation resistance ($\sigma_{c}^{\prime }$) as a function of temperature (T) and impact energy (E*) for the UNS S32750 SDSS alloy.

Figure 7 illustrates the evolution of the deformation resistance ($\sigma_{c}^{\prime }$) as a function of temperature (T) and impact energy (E*). Analysing the evolution of the deformation resistance ($\sigma_{c}^{\prime }$) as a function of temperature (T), it can be observed that the deformation resistance ($\sigma_{c}^{\prime }$) shows a decreasing tendency while increasing the deformation temperature for all impact energies. The highest deformation resistance ($\sigma_{c}^{\prime }$) was recorded in the case of the 1050 °C temperature, followed by the 1100 °C, 1150 °C, and 1200 °C temperatures, respectively. Similarly, analysing the evolution of the deformation resistance ($\sigma_{c}^{\prime }$) as a function of impact energy (E*), it can be observed that the deformation resistance ($\sigma_{c}^{\prime }$) shows an increasing tendency while increasing the impact energy (E*) for all deformation temperatures. The highest deformation resistance ($\sigma_{c}^{\prime }$) was recorded for the 545.2 J impact energy, followed by 1021.5 J, 1480.6 J, and 1905.3 J, respectively.

By analysing the rate of increase in the deformation resistance ($\sigma_{c}^{\prime }$) as a function of the applied impact energy, one can notice that the rate of increase in the deformation resistance is approximately constant while the increase in the applied impact energy, i.e., the rate of increase in the deformation resistance between 545.2 J and 1905.3 J for a deformation temperature of 1050 °C, is situated close to 0.0258 MPa/J, while for a deformation temperature of 1200 °C, it is situated close to 0.0266 MPa/J.

**Figure 8:**
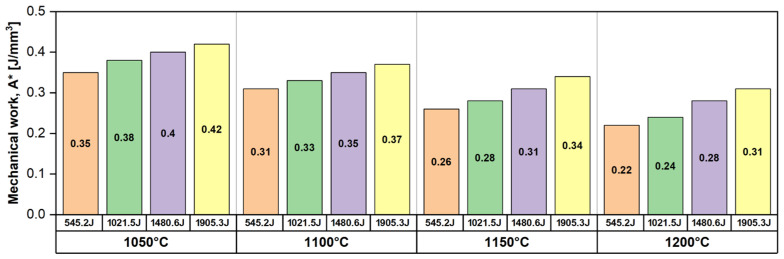
The evolution of mechanical work (A*) as a function of temperature (T) and impact energy (E*) for the UNS S32750 SDSS alloy.

Figure 8 illustrates the evolution of mechanical work ($A^{*}$) as a function of temperature (T) and impact energy (E*). When analysing the evolution of the mechanical work ($A^{*}$) as a function of temperature (T), it can be observed that the mechanical work ($A^{*}$) shows a decreasing tendency while increasing the deformation temperature for all impact energies. The highest mechanical work ($A^{*}$) was recorded in the case of the 1050 °C temperature, followed by the 1100 °C, 1150 °C, and 1200 °C temperatures, respectively. Similarly, analysing the evolution of the mechanical work ($A^{*}$) as a function of impact energy (E*), it can be observed that the mechanical work ($A^{*}$) shows an increasing tendency while increasing the impact energy (E*) for all deformation temperatures. The highest mechanical work ($A^{*}$) was recorded for the 545.2 J impact energy, followed by 1021.5 J, 1480.6 J, and 1905.3 J, respectively.

By analysing the rate of increase in the mechanical work ($A^{*}$) as a function of the deformation temperature, one can notice that the rate of increase in mechanical work shows a small decrease, i.e., the rate of increase in the mechanical work between a deformation temperature of 1050 °C and 1200 °C for an impact energy of 545.2 J is situated close to 8.7 × 10^−4^ J/mm^3^·°C, while for an impact energy of 1905.3 J, it is situated close to 7.3 × 10^−4^ J/mm^3^·°C, which is a decrease of about 15%.

Based on the analysis of the obtained deformation degree (degree of reduction in height) (ε), the deformation resistance ($\sigma_{c}^{\prime }$), and the mechanical work ($A^{*}$) evolutions as a function of temperature (T), one can observe that the obtained deformation degree (degree of reduction in height) (ε) is increasing, while both deformation resistance ($\sigma_{c}^{\prime }$) and the mechanical work ($A^{*}$) are decreasing with the deformation temperature increasing. When all are analysed as a function of impact energy (E*), one can observe that all are increasing with the impact energy (E*) increasing.

Overall, based on the data presented in Figure 6, Figure 7 and Figure 8, one can assume that the investigated UNS S32750 SDSS alloy shows a low deformation resistance and a high plasticity when deformed at temperatures between 1050 °C and 1200 °C. (i.e., 1050 °C → $\sigma_{c}^{\prime }$ ≅ 357 MPa; ε ≅ 26% and 1200 °C → $\sigma_{c}^{\prime }$ ≅ 236 MPa; ε ≅ 65%).

#### 3.2.2. Microstructure Evolution During Hot-Rolling Deformation

Based on the deformability obtained data, it was chosen to investigate the microstructural evolution during hot-rolling deformation performed in the following processing conditions:

- Applied deformation degree: 40%, 60%, and 70%;
- Applied deformation temperature: 1050 °C, 1100 °C, 1150 °C, and 1200 °C.

**Figure 9:**
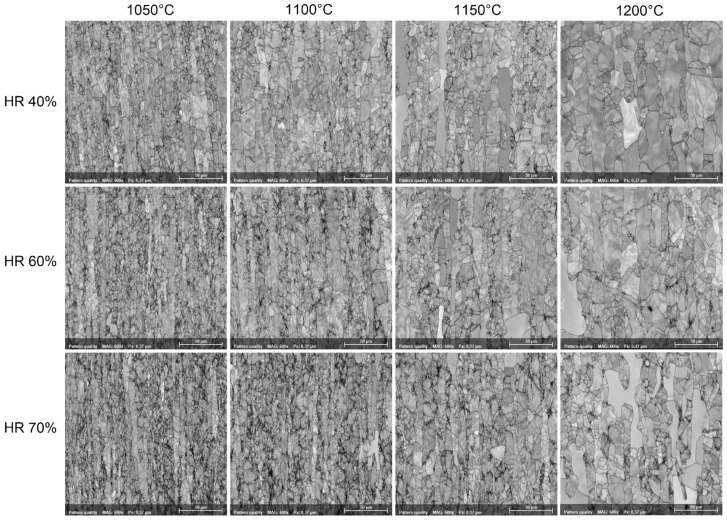
Typical SEM-EBSD microstructural images of hot-rolled (HR) UNS S32750 SDSS alloy as a function of deformation temperature (T) and applied deformation degree (ε).

Figure 9 shows typical SEM-EBSD microstructural images of hot-deformed by rolling (HR—hot-rolled) UNS S32750 SDSS alloy as a function of deformation temperature (T) and applied deformation degree (ε). One can observe firstly, at lower deformation temperatures, that the microstructure shows intensely deformed grains, strongly elongated in the rolling direction in both the ferrite (δ) and austenite (γ) grain cases. Secondly, the intensity of grain deformation is increasing with the increase in the applied deformation degree from 40% to 70%. Thirdly, increasing the deformation temperature from 1050 °C to 1200 °C leads to a strong increase in the weight fraction of the dynamically recrystallised (DRX) grains. All observations suggest that during TM processing, two main phenomena are occurring simultaneously, firstly, represented by the deformation accommodation mechanism, which accommodates the applied deformation at grain level by slip/twinning, and secondly, represented by the dynamic recrystallisation of the deformed grains. Both mechanisms occur simultaneously but with different intensities, depending on the applied deformation and temperature.

**Figure 10:**
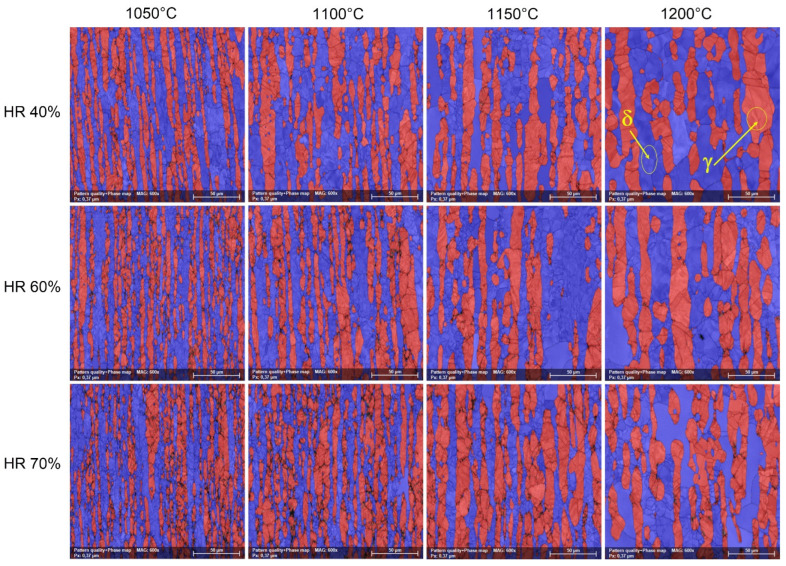
Typical SEM-EBSD phase distribution maps of the hot-rolled (HR) UNS S32750 SDSS alloy as a function of deformation temperature (T) and applied deformation degree (ε).

Figure 10 shows typical SEM-EBSD phase distribution maps of hot-deformed by rolling (HR—hot-rolled) UNS S32750 SDSS alloy as a function of deformation temperature (T) and applied deformation degree (ε). One can observe that in all cases the microstructure consists only of ferrite (δ) and austenite (γ) phases. The phase distribution maps show that the austenite (γ) phase grains are strongly elongated in the rolling direction within a matrix consisting of the ferrite (δ) phase. No other secondary phases were observed. The wight-fraction measurements of the constituent phases showed minimal changes in comparison with the as-received (AR) state.

**Figure 11:**
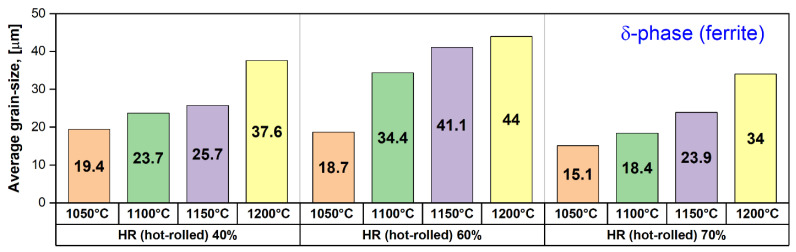
Average grain size evolution of the ferrite (δ) phase in the hot-rolled (HR) UNS S32750 SDSS alloy as a function of deformation temperature (T) and applied deformation degree (ε).

Figure 11 shows the evolution of the average grain size of the ferrite (δ) phase as a function of the deformation temperature (T) and applied deformation degree (ε). Generally, one can observe that the average grain size of the ferrite (δ) phase is increasing with the deformation temperature increasing. Firstly, applying a deformation of 40% leads to an average grain size increase from 19.4 μm for a deformation temperature of 1050 °C to 37.6 μm for a deformation temperature of 1200 °C, which represents a rate of increase of approximately 0.121 μm/°C. Secondly, increasing the applied deformation to 60% leads to an average grain size increase from 18.7 μm for a deformation temperature of 1050 °C to 44 μm for a deformation temperature of 1200 °C, which represents a rate of increase of approximately 0.168 μm/°C. Thirdly, increasing the applied deformation to 70% leads to an average grain size increase from 15.1 μm for a deformation temperature of 1050 °C to 34 μm for a deformation temperature of 1200 °C, which represents a rate of increase of approximately 0.126 μm/°C.

All of these observations confirm that the intensity of the deformation accommodation at the ferrite (δ) phase grains level by slip/twinning is increasing, while the applied deformation is increasing, leading to an intense grain refining. At the same time, the dynamic recrystallisation of the deformed ferrite (δ) phase grains occurs, leading to the generation of new ferrite (δ) phase grains, which are showing an increased growth rate due to the increased deformation temperature.

**Figure 12:**
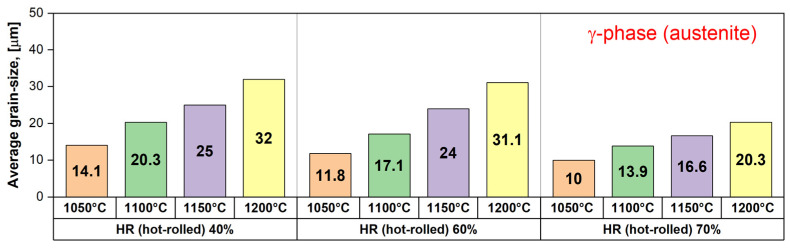
Average grain size evolution of the austenite (γ) phase in the hot-rolled (HR) UNS S32750 SDSS alloy as a function of deformation temperature (T) and applied deformation degree (ε).

Figure 12 shows the evolution of the average grain size of the austenite (γ) phase as a function of the deformation temperature (T) and applied deformation degree (ε). Generally, one can observe that the average grain size of the austenite (γ) phase is increasing with the deformation temperature increasing. Firstly, applying a deformation of 40% leads to an average grain size increase from 14.1 μm for a deformation temperature of 1050 °C to 32 μm for a deformation temperature of 1200 °C, which represents a rate of increase of approximately 0.119 μm/°C. Secondly, increasing the applied deformation to 60% leads to an average grain size increase from 11.8 μm for a deformation temperature of 1050 °C to 31.1 μm for a deformation temperature of 1200 °C, which represents a rate of increase of approximately 0.128 μm/°C. Thirdly, increasing the applied deformation to 70% leads to an average grain size increase from 10 μm for a deformation temperature of 1050 °C to 20.3 μm for a deformation temperature of 1200 °C, which represents a rate of increase of approximately 0.068 μm/°C. All of these observations confirm that the intensity of the deformation accommodation at the austenite (γ) phase grain level by slip/twinning is increasing, while the applied deformation is increasing, leading to an intense grain refining. At the same time, the dynamic recrystallisation of the deformed austenite (γ) phase grains is occurring, but with a much lower intensity in comparison with the one observed in the case of the ferrite (δ) phase, the reason for which the obtained grain size in the case of the austenite (δ) phase is much lower.

Comparing the obtained data in the case of the austenite (γ) phase with the one obtained in the case of the ferrite (δ) phase, one can observe that the austenite (γ) phase accommodates better the applied deformation due to the specificity of the FCC (face-centred cubic) crystalline system of the austenite (γ) phase in comparison with the BCC (body-centred cubic) crystalline system of the ferrite (δ) phase, which possesses a superior number of active slip/twinning systems. Also, it can be observed that the intensity of the dynamic recrystallisation of the ferrite (δ) phase is higher in comparison with the one observed in the case of the austenite (γ) phase.

**Figure 13:**
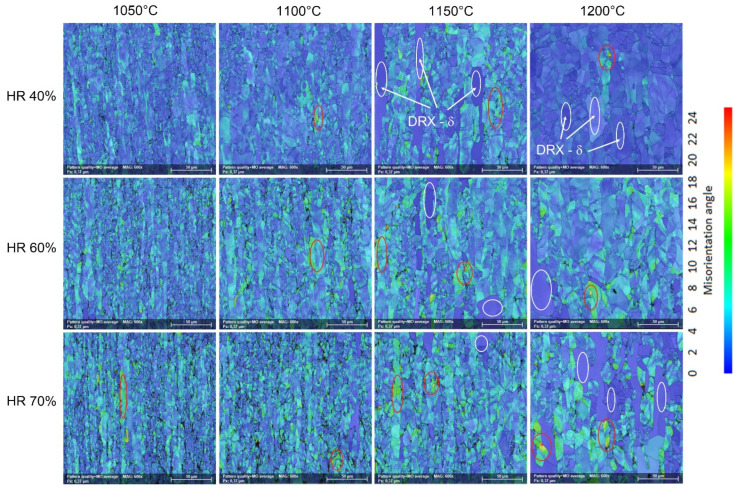
Typical SEM-EBSD Modal Orientation (MO) distribution maps of the hot-rolled (HR) UNS S32750 SDSS alloy as a function of deformation temperature (T) and applied deformation degree (ε).

Figure 13 shows the evolution of the Modal Orientation (MO) distribution maps as a function of the deformation temperature (T) and applied deformation degree (ε). When analysing the evolution of the MO, one can observe the following:

Firstly, increasing the applied deformation leads to an increase in the maximum misorientation of the constituent ferrite (δ) and austenite (γ) phase grains, with higher values being observed in the case of the ferrite (δ) phase grains. Applying a 40% deformation leads to a maximum misorientation observed in the ferrite (δ) phase grains close to 18°; increasing the applied deformation to 60% leads to a maximum misorientation close to 22°; and, finally, increasing the applied deformation to 70% leads to a maximum misorientation close to 24°. All of this shows that the austenite (γ) phase accommodates better the applied deformation due to its superior number of active slip/twinning systems.

Secondly, increasing the deformation temperature leads to an increase in the intensity of the dynamic recrystallisation of deformed ferrite (δ) and austenite (γ) phase grains. It can be observed that by increasing the deformation temperature, the number of dynamically recrystallised (DRX) ferrite (δ) phase grains is increasing (which is characterised by a low misorientation, usually below 4°). Also, at the same time, it can be observed that the average grain size of the dynamic recrystallised (DRX) ferrite (δ) phase grains is increasing.

## 4. Conclusions

The high-temperature deformation behaviour of the UNS S32750 Super Duplex Stainless Steel (SDSS) alloy can be summarised as follows:

- Increasing the deformation degree (degree of reduction in height) can be achieved by increasing the deformation temperature.
- Increasing the applied impact energy can lead to an increase in the deformation degree.
- The evolution of the deformation resistance ($\sigma_{c}^{\prime }$) as a function of temperature (T) shows a decreasing tendency while increasing the deformation temperature for all impact energies. The evolution of the mechanical work ($A^{*}$) as a function of temperature (T) shows a decreasing tendency while increasing the deformation temperature for all impact energies.
- In all cases, the microstructure shows intensely deformed grains, strongly elongated in the rolling direction in both the ferrite (δ) and austenite (γ) intensely deformed grains. The intensity of the grain deformation is increasing with the increase in the applied deformation degree. Also, it was observed that increasing the deformation temperature leads to a strong increase in the weight fraction of the dynamically recrystallised (DRX) ferrite (δ) grains.

Further investigation of the high-temperature deformation behaviour of the UNS S32750 SDSS alloy is currently being undertaken by the present authors in order to fully understand the occurring phenomena during deformation.

## Figures and Tables

**Table 1:** The evolution of deformation degree (ε) as a function of strike height (H), impact energy (E*), and temperature (T) for the UNS S32750 SDSS alloy.

Hammer mass, m [kg]	117			
Strike height, H [m]	0.5	1.0	1.5	2.0
Impact energy, E* [J]	545.2	1021.5	1480.6	1905.3
Up-setting temperature, T [°C]	1050			
Obtained deformation degree, ε [%]	16.52	28.77	39.67	48.06
Up-setting temperature, T [°C]	1100			
Obtained deformation degree, ε [%]	18.73	33.10	44.41	53.94
Up-setting temperature, T [°C]	1150			
Obtained deformation degree, ε [%]	22.18	38.30	50.74	59.28
Up-setting temperature, T [°C]	1200			
Obtained deformation degree, ε [%]	26.16	44.31	55.79	64.83

**Table 2:** The evolution of deformation resistance ($\sigma_{c}^{\prime }$) and mechanical work ($A^{*}$) as a function of temperature (T) and impact energy (E*) for the UNS S32750 SDSS alloy.

Up-setting temperature, T [°C]	1050	1100	1150	1200
Impact energy, E* [J]	545.2			
$\mathrm{Deformation}\;\mathrm{resistance},\;\sigma_{c}^{\prime }$ [MPa]	321.9	283.1	237.7	200.1
$\mathrm{Mechanical}\;\mathrm{work},\;A^{*}$ [J/mm^3^]	0.35	0.31	0.26	0.22
Impact energy, E* [J]	1021.5			
$\mathrm{Deformation}\;\mathrm{resistance},\;\sigma_{c}^{\prime }$ [MPa]	339.2	291.9	248.8	210.8
$\mathrm{Mechanical}\;\mathrm{work},\;A^{*}$ [J/mm^3^]	0.38	0.33	0.28	0.24
Impact energy, E* [J]	1480.6			
$\mathrm{Deformation}\;\mathrm{resistance},\;\sigma_{c}^{\prime }$ [MPa]	346.7	304.7	259.5	229.5
$\mathrm{Mechanical}\;\mathrm{work},\;A^{*}$ [J/mm^3^]	0.40	0.35	0.31	0.28
Impact energy, E* [J]	1905.3			
$\mathrm{Deformation}\;\mathrm{resistance},\;\sigma_{c}^{\prime }$ [MPa]	357.1	308.9	271.4	236.3
$\mathrm{Mechanical}\;\mathrm{work},\;A^{*}$ [J/mm^3^]	0.42	0.37	0.34	0.31

## Data Availability

The raw data supporting the conclusions of this article will be made available by the authors on request.
